# Signaling pathways activation profiles make better markers of cancer than expression of individual genes

**DOI:** 10.18632/oncotarget.2548

**Published:** 2014-08-23

**Authors:** Nikolay M. Borisov, Nadezhda V. Terekhanova, Alexander M. Aliper, Larisa S. Venkova, Philip Yu Smirnov, Sergey Roumiantsev, Mikhail B. Korzinkin, Alex A. Zhavoronkov, Anton A. Buzdin

**Affiliations:** ^1^ Pathway Pharmaceuticals, Wan Chai, Hong Kong, Hong Kong SAR; ^2^ Laboratory of Systems Biology, A.I. Burnasyan Federal Medical Biophysical Center, Moscow, 123182, Russia; ^3^ Laboratory of Bioinformatics, D. Rogachyov Federal Research Center of Pediatric Hematology, Oncology and Immunology, Moscow, 117198, Russia; ^4^ Group for Genomic Regulation of Cell Signaling Systems, Shemyakin-Ovchinnikov Institute of Bioorganic Chemistry, Moscow, 117997, Russia

**Keywords:** Cancer, Intracellular signaling pathway activation, Gene expression, Transcriptome profiling, Molecular markers, AUC

## Abstract

Identification of reliable and accurate molecular markers remains one of the major challenges of contemporary biomedicine. We developed a new bioinformatic technique termed OncoFinder that for the first time enables to quantatively measure activation of intracellular signaling pathways basing on transcriptomic data. Signaling pathways regulate all major cellular events in health and disease. Here, we showed that the Pathway Activation Strength (PAS) value itself may serve as the biomarker for cancer, and compared it with the “traditional” molecular markers based on the expression of individual genes. We applied OncoFinder to profile gene expression datasets for the nine human cancer types including bladder cancer, basal cell carcinoma, glioblastoma, hepatocellular carcinoma, lung adenocarcinoma, oral tongue squamous cell carcinoma, primary melanoma, prostate cancer and renal cancer, totally 292 cancer and 128 normal tissue samples taken from the Gene expression omnibus (GEO) repository. We profiled activation of 82 signaling pathways that involve ~2700 gene products. For 9/9 of the cancer types tested, the PAS values showed better area-under-the-curve (AUC) scores compared to the individual genes enclosing each of the pathways. These results evidence that the PAS values can be used as a new type of cancer biomarkers, superior to the traditional gene expression biomarkers.

## INTRODUCTION

Identification of reliable and accurate molecular markers of cancer remains one the major challenges of contemporary biomedicine. Thousands of reports have been published communicating new RNA, protein and non-protein biochemical biomarkers sensitive to cancer development [1–7]. Most of these markers represent products of individual gene expression at the RNA or protein levels. Some of them are widely used in clinical practice, but there remains an overall unsolved problem of finding new cancer biomarkers with enhanced specificity and sensitivity scores compared to the existing ones. Another aspect of the same problem deals with the shortage of the cancer type-specific molecular markers, e.g. melanoma-specific, bladder or pancreatic cancer-specific, etc. Association of the marker expression with the success of the medical treatment may provide clues to a more efficient, patient-oriented cancer treatment therapy [8].

Recently, we developed a new bioinformatic technique termed OncoFinder. This novel program enables the user to quantatively measure the activation of intracellular signaling pathways in a number of cell/tissue physiological and pathological conditions including cancer. Signaling pathways regulate all major cellular events in health and disease [9–11].

OncoFinder takes transcriptome-wide gene expression levels, including microarray and next-generation sequencing (NGS) data as input and calculates a quantitative measure of the signaling pathway activation strength (*PAS*) for the signaling pathways under investigation. The *PAS* is a measure of the cumulative value of perturbations of a signaling pathway and it may serve as a distinct indicator of pathological changes in the intracellular signalization machine at the cellular, tissue or organ levels.

The formula for *PAS* calculations include gene expression data and the information of the protein interactions in the pathway under investigation, namely, the protein activator or repressor of the pathway [12]; for the pathway *p*, $PAS_{p}=\sum ARR_{np}\cdot \mathrm{lg}(CNR_{n})$. Here the summation is done over all the gene products in a pathway, which represents the signal through a pathway *p*. The relative role of a gene product in signal transduction is reflected by a discrete flag *activator/repressor role* (*ARR*) which equals to 1 for an activator gene product; −1 for a repressor, and intermediate values −0,5; 0,5 and 0 for the gene products that have rather repressor, activator or unknown roles, respectively. The *CNR_n_* value (*case-to-normal ratio)* is the ratio of the expression level of a gene *n* in the sample under investigation, to the average expression level in the sampling used as the norm for this comparison. The positive value of PAS indicates abnormal activation of a signaling pathway, and the negative value – its repression. With the exception of pediatric oncology, the majority of cancers are age-related [13]. The methods for calculating PAS, CNR and the drug score in cancer were proposed in the study of aging [12, 14]. In the investigations with the experimentally-tracked data on the signaling pathway activation, we have previously confirmed the robustness of this formula and its adequacy to the analysis of intracellular signalization [12]. The above formula for PAS calculation was shown to dramatically diminish the discrepencies between the microarray and deep sequencing data obtained using various experimental platforms [15].

Calculations were made that take into account the relative importance of certain genes and their products according to the results of parameter sensitivity [16] and/or stiffness/sloppiness analysis [17] in terms of total concentrations of certain proteins using an approved kinetic model of signaling pathway activation [18].

Here, we investigated if the *PAS* value itself may serve as the biomarker for cancer, and compared it with “traditional” molecular markers based on the expression of individual genes. We applied OncoFinder to gene expression datasets for the nine human cancer types including bladder cancer, basal cell carcinoma, glioblastoma, hepatocellular carcinoma, lung adenocarcinoma, oral tongue squamous cell carcinoma, primary melanoma, prostate cancer and renal cancer. This covers 292 cancer and 128 normal tissue samples from the Gene expression omnibus (GEO) repository [19]. We profiled the activation of 82 signaling pathways that involve ~2700 individual gene products. For 9/9 of these cancer types, the SPA values showed significantly better area-under-the-curve (AUC) scores compared to the individual genes enclosing each of the pathways. These results provide evidence that the SPA values calculated using OncoFinder algorithm can be used as a new type of cancer biomarkers, superior to the traditional gene expression biomarkers.

## RESULTS AND DISCUSSION

### Profiling pathway activation strength *(PAS)* for cancer transcriptomes

Using the recently published algorithm for calculating *PAS* values [12] we profiled the large-scale transcriptomic data obtained for the nine types of human cancer and for the matching normal tissues (Table 1). In total we analized 292 cancer and 128 matching normal transcriptomes from the Gene expression omnibus (GEO) repository. This covered the following cancers; bladder cancer, basal cell carcinoma, glioblastoma, hepatocellular carcinoma, lung adenocarcinoma, oral tongue squamous cell carcinoma, primary melanoma, prostate cancer and renal cancer. All the transcriptomic datasets were synthesized using the same microarray platform Affymetrix Human Genome U133 Plus 2.0 [20–27].

**Table 1:** Transcriptomic datasets extracted from the GEO repository

Cancer type	Number of cancer samples	Number of normal samples	Reference	GEO dataset number
Basal cell carcinoma	15	4	[19]	GSE7553
Bladder cancer	52	40	[20]	GSE31189
Glioblastoma	34	13	[21]	GSE50161
Hepatocellular carcinoma	10	10	[22]	GSE29721
Lung adenocarcinoma	86	13	[23]	GSE30219
Oral tongue squamous cell carcinoma	26	12	[24]	GSE9844
Primary melanoma	14	4	[19]	GSE7553
Prostate cancer (well differentialed)	20	20	[25]	GSE32448
Renal cancer	35	12	[26]	GSE7023

We interrogated a total of 82 intracellular signaling pathways encompassing the products of ~2700 human genes (Supplementary dataset 1). Basing on the comparison of the cancer vs normal tissue transcriptomic data, we obtained the *PAS* profiles characteristic of the above cancer types (Supplementary datasets 2, 3). Positive and negative *PAS* scores reflect upregulated and downregulated signaling pathways, respectively, whereas zero *PAS* scores represent unaffected pathways acting similarly in cancer and in normal tissues.

**Figure 1:**
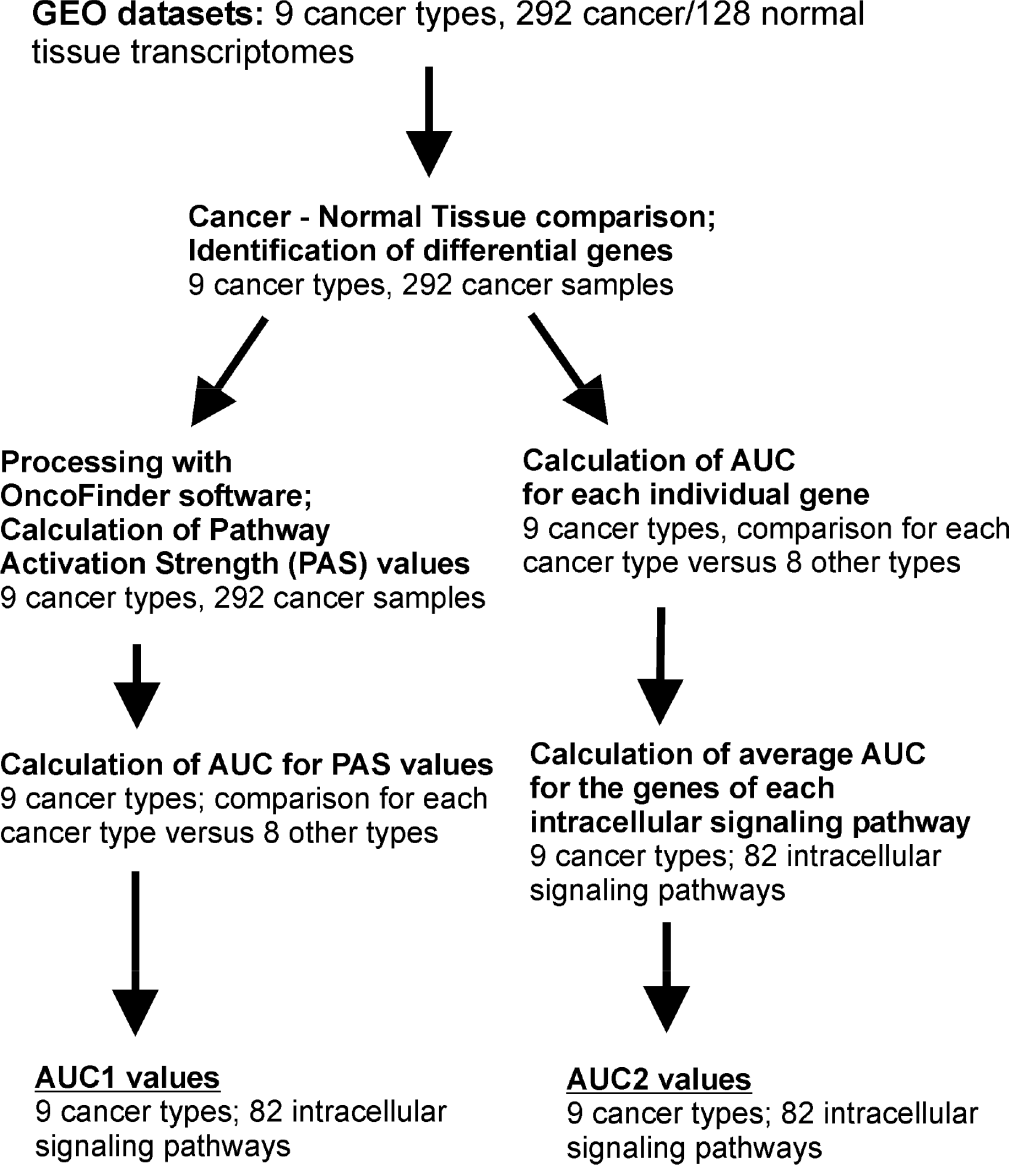
Outline of the bioinformatics procedures used to calculate AUC1 and AUC2 values

We next calculated the area-under-curve (AUC) values [28] for the *PAS* scores of each of the pathways under investigation. The AUC value is the universal characteristics of biomarker robustness and it is dependent on the sensitivity and specificity of a biomarker. It correlates positively with the biomarker quality and may vary in an interval from 0.5 till 1. The AUC threshold for discriminating good and bad biomarkers is typically 0.7 or 0.75. The entries having greater AUC score are considered good-quality biomarkers and vice-versa [29]. The AUC values were calculated when comparing each cancer type against the remaining eight cancer types. Enhanced AUC values here meant that the corresponding signaling pathway is a good biomarker distinguishing an individual cancer type from the others (Supplementary dataset 4). This kind of AUC score will be referred here as *AUC1* (Supplementary dataset 4). In parallel, we also calculated the analogous AUC scores for the individual gene products (namely, for the values of lg *CNR* for them, Supplementary dataset 5) including those involved in each signaling pathway. For these individual gene products involved in the pathways (a total of 2726 human gene products), we next calculated the *average* AUC scores characteristic of each signaling pathway/cancer type, referred here as *AUC2* (Supplementary dataset 4). The AUC2 value for pathway *p* and cancer type *n* is the average of the cancer *n*-associated AUC scores for all the gene products involved in the pathway *p*. The outline of the data analysis is shown on the Figure 1.

### Comparison of the AUC scores calculated for the pathway activation strength and for the individual gene expression levels

AUC1 reflects the quality of PAS as the biomarker for a given signaling pathway, and AUC2 is the integral characteristics of the biomarker quality for the expression of the genes which are involved in the same pathway. The results of the AUC calculations (Supplementary dataset 4) showed that among the good-quality biomarkers (AUC cut-off value 0.7 or 0.75) the values for AUC1 were higher than for the AUC2 for all cancer types (Table 2). For example, for the cut-off value 0.75 in all cancer types there were only 14 AUC2 (gene expression) markers, in contrast to 160 AUC1 (pathway activation) markers (Table 2). Moreover, for ten of these fourteen AUC2 markers, the corresponding AUC1 values were greater (Table 2), thus suggesting the stronger biomarker potential of the AUC1 (pathway activation) markers.

**Table 2:** Comparison of the AUC1 and AUC2 scores calculated for 81 intracellular signaling pathways for nine human cancer types basing on the transcriptomic data

Cancer type	AUC1 > 0.7^a^	AUC2 > 0.7^b^	AUC1/2 > 0.7; AUC1 > AUC2^c^	AUC1/2 > 0.7; AUC2 > AUC1^d^	AUC1 > 0.75^e^	AUC2 > 0.75^f^	AUC1/2 > 0.75; AUC1 > AUC2^g^	AUC1/2 > 0.75; AUC2 > AUC1^h^
Basal cell carcinoma	40	5	40	1	23	0	23	0
Bladder cancer	20	23	15	15	10	9	8	4
Glioblastoma	66	68	66	12	59	5	59	0
Hepatocellular	17	0	17	0	7	0	7	0
Lung adenocarcinoma	32	2	32	1	21	0	21	0
Oral tongue squamous cell carcinoma	5	0	5	0	2	0	2	0
Primary melanoma	25	0	25	0	13	0	13	0
Prostate cancer	28	8	28	5	16	0	16	0
Renal cancer	19	10	19	6	10	0	10	0

a Number of signaling pathways where AUC1 > 0.7

b Number of signaling pathways where AUC2 > 0.7

c Number of signaling pathways where AUC1/2 > 0.7, and AUC1 > AUC2

d Number of signaling pathways where AUC1/2 > 0.7, and AUC2 > AUC1

e Number of signaling pathways where AUC1 > 0.75

f Number of signaling pathways where AUC2 > 0.75

g Number of signaling pathways where AUC1/2 > 0.75, and AUC1 > AUC2

h Number of signaling pathways where AUC1/2 > 0.75, and AUC2 > AUC1

Importantly, these data show that the pathway activation strength (*PAS*) – based biomarkers may serve efficiently to distinguish the different cancer types. Among the 82 signaling pathways profiled in this assay, 75 showed a potential to serve as the strong cancer type-specific biomarkers with the AUC > 0.75 (Supplementary dataset 4). For each cancer type, the number of these PAS biomarkers (AUC > 0.75) varied from 2 till 59 (Table 2). The quality of these biomarkers was typically stronger than for the biomarkers purely based on the gene expression levels, as reflected by the comparison of AUC1 vs AUC2 scores (Table 2). This suggests that during cancer progression the signaling pathway regulation is a more uniform process rather than the activation of certain individual genes. Indeed, an intracellular signaling pathway is a complex regulatory network that may include hundreds of different gene products [30–31]. Theoretically, expression of every gene in this network may have an influence on the overall functioning of the signaling pathway. Alterations in the expression profiles of many different genes can, therefore, lead to a similar result of a pathway activation or suppression during cancer development [32].

In this study, we for the first time quantitatively profiled the signaling pathway activation features in 292 human cancer samples. The profiles obtained here for the bladder cancer, basal cell carcinoma, glioblastoma, hepatocellular carcinoma, lung adenocarcinoma, oral tongue squamous cell carcinoma, primary melanoma, prostate cancer and renal cancer are available in the Supplementary dataset 2. Further thourough analysis of these data is underway in our laboratory and will be published elsewhere. In this report, we want to discuss the pathway activation features that may serve to distinguish the different types of cancer (see the Supplementary dataset 6). These signaling pathways can be either upregulated or suppressed, with the characteristic values of *PAS* used as the distinguishing features. For example, with the AUC cut-off threshold 0.75, downregulation of the ATM pathway (average *PAS*~ −2) characterizes hepatocellular carcinoma, whereas its upregulation (avg *PAS*~3.7) is typical for the melanoma cells. Strong increase in Notch signaling (avg *PAS*~9) denotes glioma, mild upregulation of RNA polymerase II complex activity (avg *PAS*~1.4) – basal cell carcinoma, moderate decrease in IP3 signaling (avg *PAS*~ −1.9) – lung adenocarcinoma, et cetera. It may be seen that any investigated tissue type has its unique profile of statistically significant pathway activation features, which provides a potent instrument for further analysis and specific targeting of various cancer types in the future.

It was shown previously that many intracellular signaling pathways actively participate in tumorigenesis [33–36]. Other pathways, in turn, are silenced in the transformed cells and tissues [37–38]. Intracellular regulation is also implicated in metastasing, drug resistance and tumor invasiveness [39–42]. We propose that the current bioinformatic approach based on the OncoFinder algorithm opens broad perspectives for finding tight associations of signaling pathway activation with the prognosis of disease progression and with the efficiency of anticancer treatment.

## CONCLUSION

In this study, we provide evidence that the signaling pathway activation strength (*PAS*) values may serve as the biomarkers of different cancer types, frequently superior than the traditional molecular markers based on the expression of individual genes. We applied our original bioinformatical algorithm OncoFinder to gene expression datasets for the nine human cancer types. This includes 292 cancer and 128 normal tissue samples taken from the Gene expression omnibus (GEO) repository. We profiled the activation of 82 signaling pathways that involve ~2700 individual gene products. For 9/9 of the cancer types, the SPA values showed significantly stronger area-under-the-curve (AUC) scores compared to the individual genes whose products are involved in the respective pathways. These results show that the SPA values calculated using OncoFinder algorithm may be used as a new type of cancer biomarker, superior to the traditional gene expression biomarkers. We also, for the first time, publish characteristic intracellular signaling pathway activation profiles for nine human types of cancer.

## METHODS

### Source datasets

Gene expression data used in this study were downloaded from the Gene Expression Omnibus (GEO) repository of transcriptomic information (http://www.ncbi.nlm.nih.gov/geo/). All the dataset were obtained using the microarray platform Affymetrix Human Genome U133 Plus 2.0 Array. The datasets used are listed on the Table 1. The normal tissue samples from each dataset were used as the controls in further calculations.

The signalome knowledge base developed by SABiosciences (http://www.sabiosciences.com/pathwaycentral.php) was used to determine structures of intracellular pathways, which was used for the computational algorithm OncoFinder exactly as described previously [12, 14, 43]. For all the datasets, the data on gene expression levels for cancer and normal tissues were quantile-normalized. For each cancer sample, the logarithmic value of lg *CNR* was used for every gene in further calculations.

### Functional annotation of gene expression data

We applied our original algorithm OncoFinder [12] for the functional annotation of the primary expression data and for the calculation of the PAS scores. The extracted raw microarray expression data were quantile normalized according to [44]. Our approach to the transcriptome-wide gene expression analysis applies processing of these results with the signalome knowledge base developed by SABiosciences (http://www.sabiosciences.com/pathwaycentral.php). The algorithm utilizes a scheme that takes into account the overall impact of each gene product in the signaling pathway but ignores its position in the pathway graph. The formula used to calculate the *pathway activation strength* (*PAS*) for a given sample and a given pathway *p* is as follows:

$$PAS_{p}=\sum_{n}ARR_{np}\cdot BTIF_{n}\cdot \mathrm{lg}(CNR_{n})$$

Here the *case-to-normal ratio*, *CNR_n_*, is the ratio of expression levels for a gene *n* in the sample under investigation to the same average value for the control group of samples. The Boolean flag of *BTIF* (*beyond tolerance interval flag*) equals zero when the *CNR* value has passed simultaneously the two criteria that demark the significantly perturbed expression level from essentially normal. The first criterion is the expression level for the sample lies within the tolerance interval, where p > 0.05. The second criterion is the discrete value of *ARR* (*activator/repressor role*) equals to the following fixed values: −1, when the gene/protein *n* is a repressor of pathway excitation; 1, if the gene/protein *n* is an activator of pathway excitation; 0, when the gene/protein *n* can be both an activator and a repressor of the pathway; 0.5 and −0.5, respectively, if the gene/protein *n* is rather an activator or repressor of the signaling pathway *p*, respectively. The results for the 82 pathways were obtained for each sample (listed in the Supplementary dataset 2). The area-under-curve (AUC) values were calculated according to [28]. Statistical tests were done using the R software package.

## SUPPLEMENTARY FIGURES AND TABLES












