# Supplementary material for: Signaling pathways activation profiles make better markers of cancer than expression of individual genes
Source: Oncotarget. 2014 Aug 23;5(20):10198–205. doi: 10.18632/oncotarget.2548 (PMC4259415; doi:10.18632/oncotarget.2548)
Supplement: Supplementary file 1 [file oncotarget-05-10198-s001.pdf]

SUPPLEMENTARY DATA

Supplementary Dataset 3: Pathway Activation Strength (PAS) for the investigated cancer types profiled for 82 human intracellular signaling pathways. Average PAS scores are shown for each cancer type.

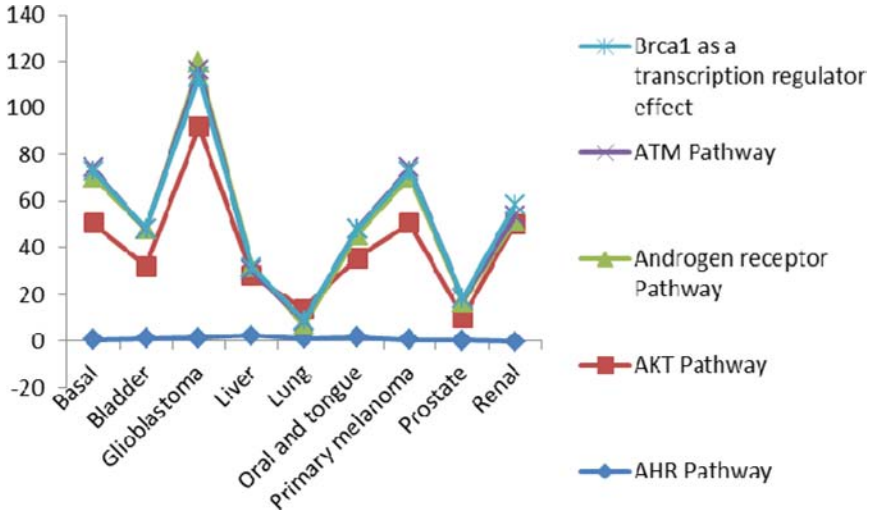

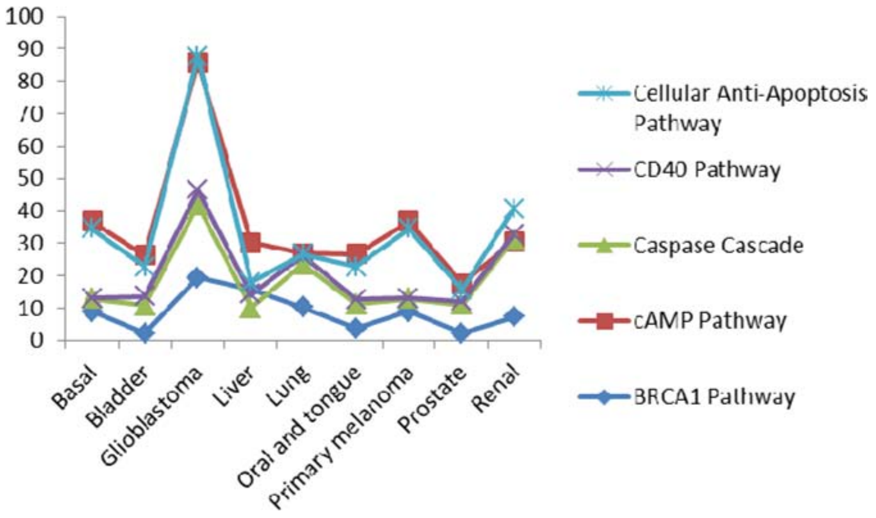

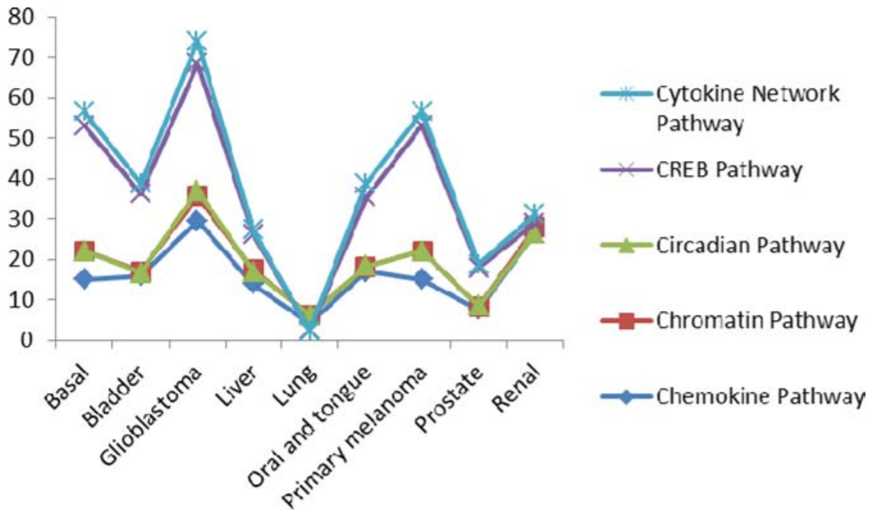

(*Continued*)

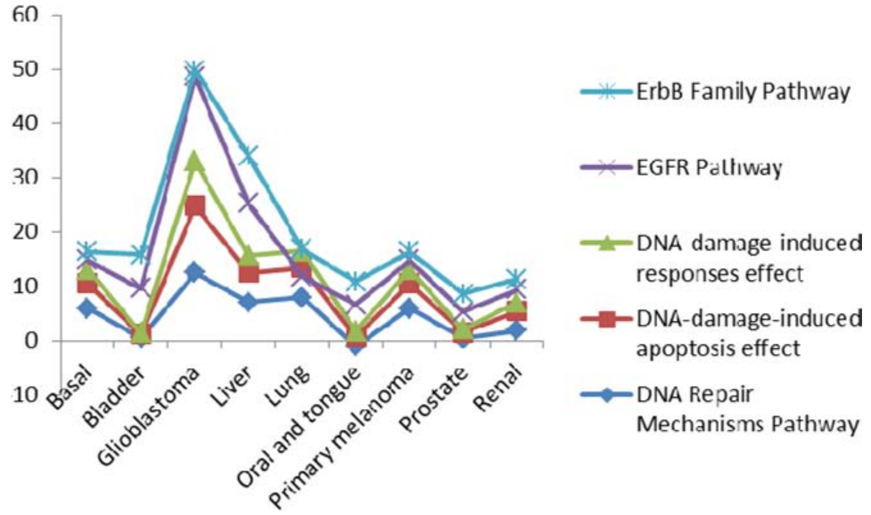
60
50
40
30
20
10
0
10
Basal
Bladder
Glioblastoma
Liver
Lung
Oral and tongue
Primary melanoma
Prostate
Renal
ErbB Family Pathway
EGFR Pathway
DNA damage induced responses effect
DNA-damage-induced apoptosis effect
DNA Repair Mechanisms Pathway

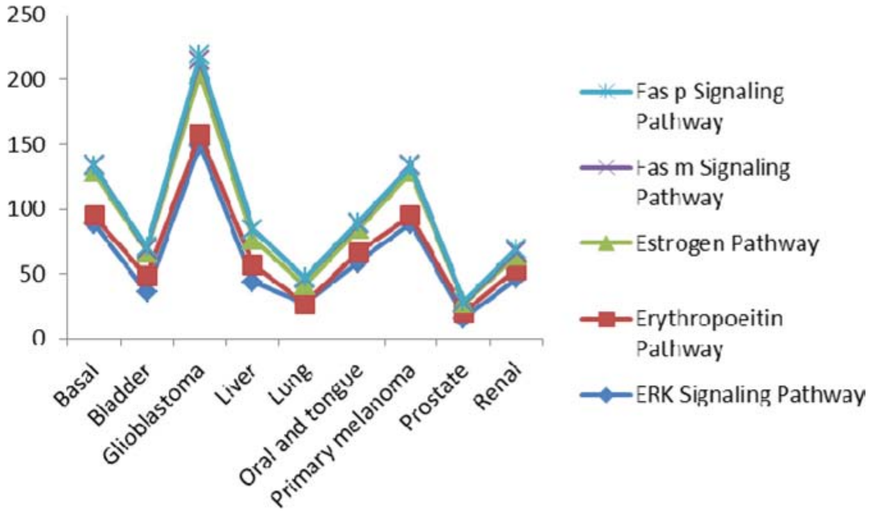
250
200
150
100
50
0
Basal
Bladder
Glioblastoma
Liver
Lung
Oral and tongue
Primary melanoma
Prostate
Renal
Fas p Signaling Pathway
Fas m Signaling Pathway
Estrogen Pathway
Erythropoeitin Pathway
ERK Signaling Pathway

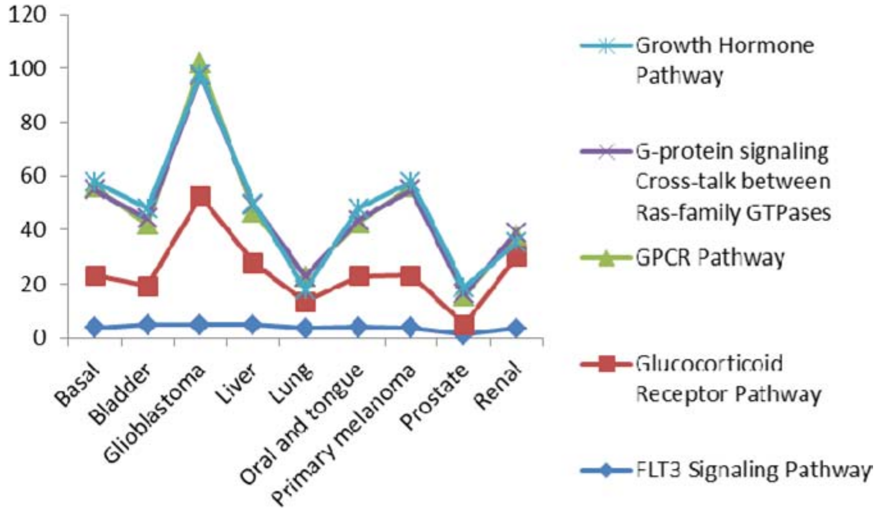
120
100
80
60
40
20
0
Basal
Bladder
Glioblastoma
Liver
Lung
Oral and tongue
Primary melanoma
Prostate
Renal
Growth Hormone Pathway
G-protein signaling Cross-talk between Ras-family GTPases
GPCR Pathway
Glucocorticoid Receptor Pathway
FLT3 Signaling Pathway

(Continued)

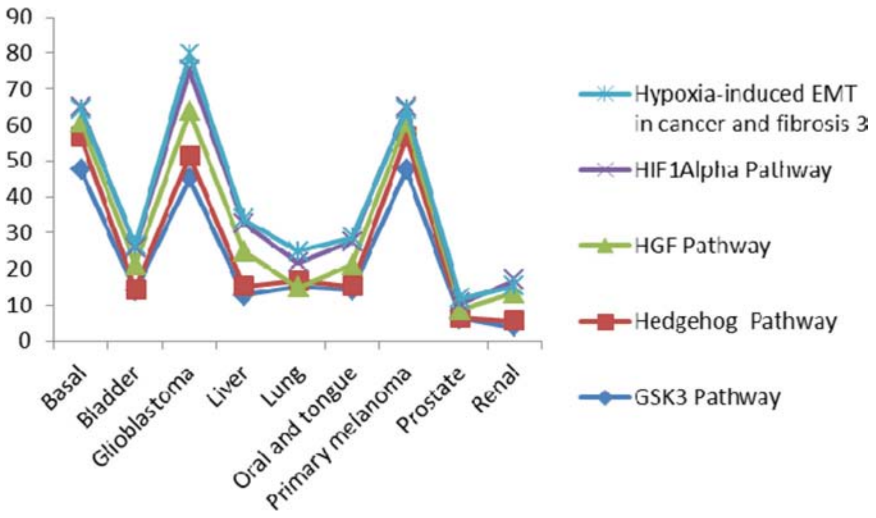
90
80
70
60
50
40
30
20
10
0
Basal
Bladder
Glioblastoma
Liver
Lung
Oral and tongue
Primary melanoma
Prostate
Renal
Hypoxia-induced EMT in cancer and fibrosis 3
HIF1Alpha Pathway
HGF Pathway
Hedgehog Pathway
GSK3 Pathway

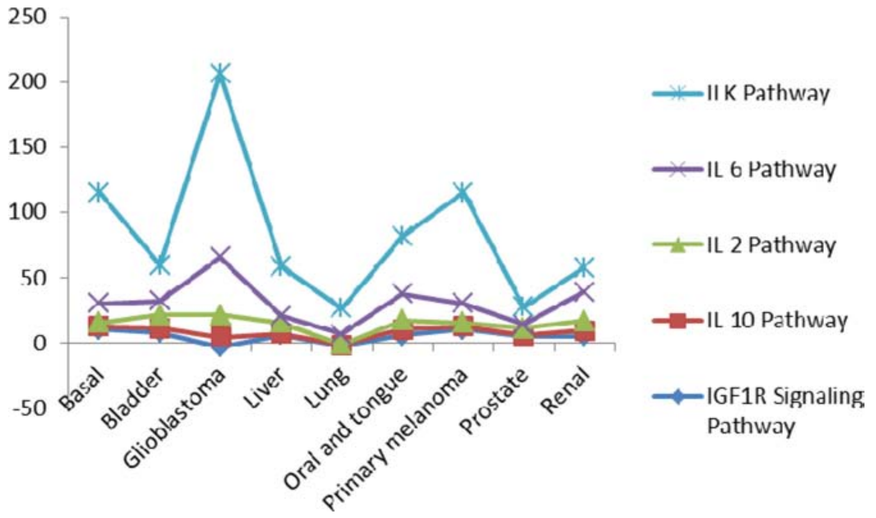
250
200
150
100
50
0
-50
Basal
Bladder
Glioblastoma
Liver
Lung
Oral and tongue
Primary melanoma
Prostate
Renal
ILK Pathway
IL 6 Pathway
IL 2 Pathway
IL 10 Pathway
IGF1R Signaling Pathway

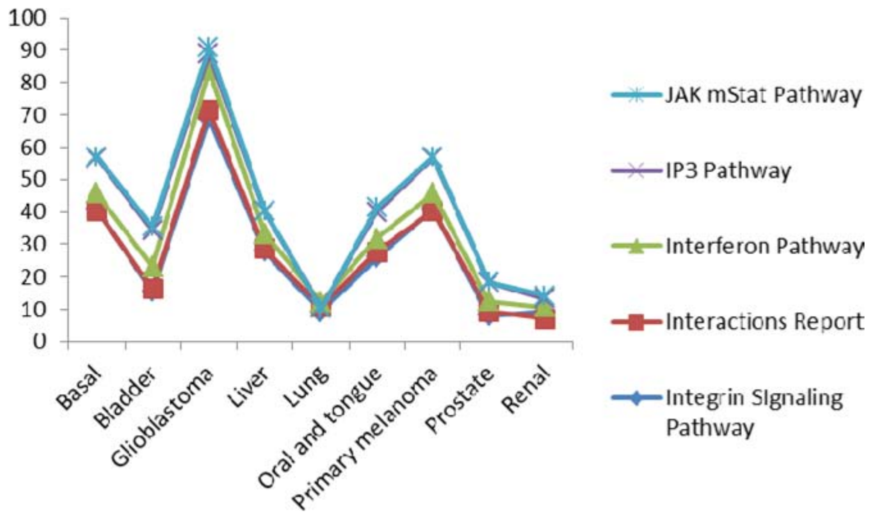
100
90
80
70
60
50
40
30
20
10
0
Basal
Bladder
Glioblastoma
Liver
Lung
Oral and tongue
Primary melanoma
Prostate
Renal
JAK mStat Pathway
IP3 Pathway
Interferon Pathway
Interactions Report
Integrin Signaling Pathway

(*Continued*)

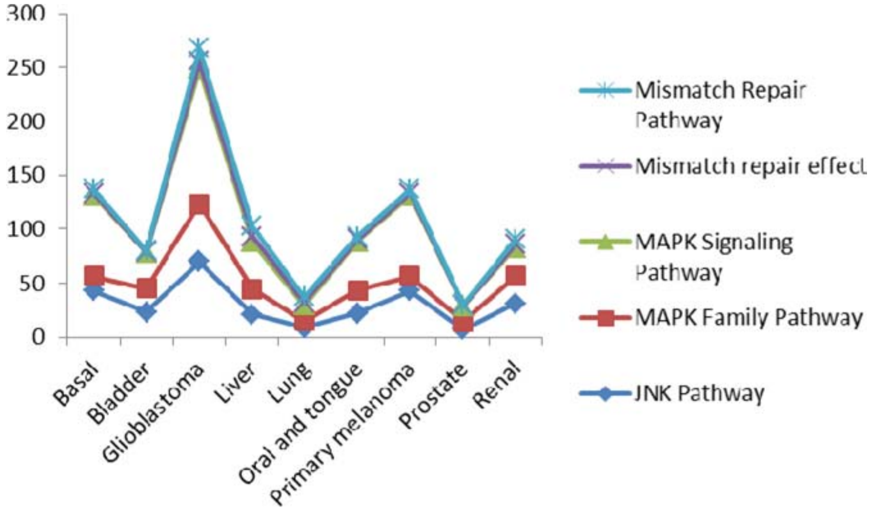
300
250
200
150
100
50
0
Basal
Bladder
Glioblastoma
Liver
Lung
Oral and tongue
Primary melanoma
Prostate
Renal
Mismatch Repair Pathway
Mismatch repair effect
MAPK Signaling Pathway
MAPK Family Pathway
JNK Pathway

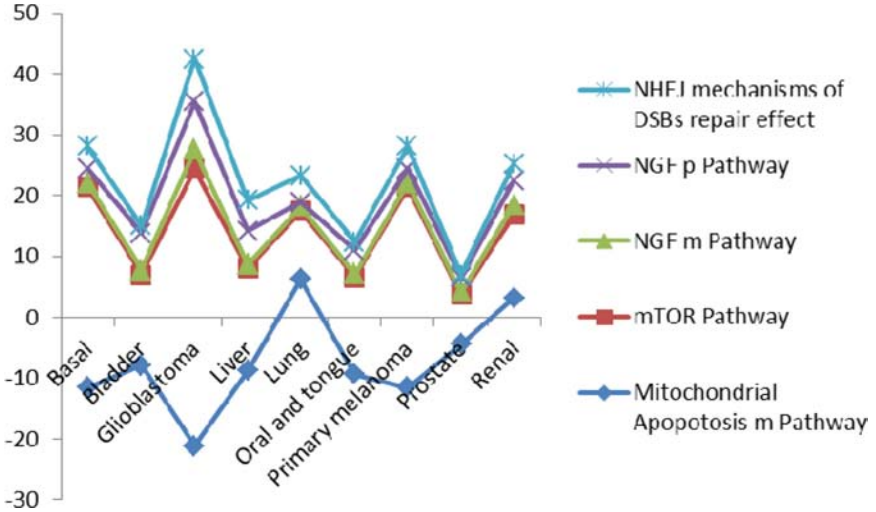
50
40
30
20
10
0
-10
-20
-30
Basal
Bladder
Glioblastoma
Liver
Lung
Oral and tongue
Primary melanoma
Prostate
Renal
NHEJ mechanisms of DSBs repair effect
NGF p Pathway
NGF m Pathway
mTOR Pathway
Mitochondrial Apopotosis m Pathway

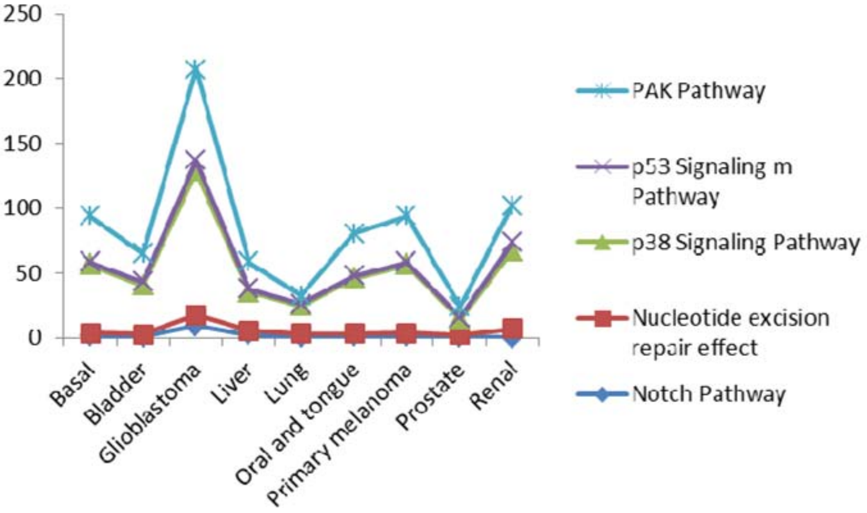
250
200
150
100
50
0
Basal
Bladder
Glioblastoma
Liver
Lung
Oral and tongue
Primary melanoma
Prostate
Renal
PAK Pathway
p53 Signaling m Pathway
p38 Signaling Pathway
Nucleotide excision repair effect
Notch Pathway

(*Continued*)

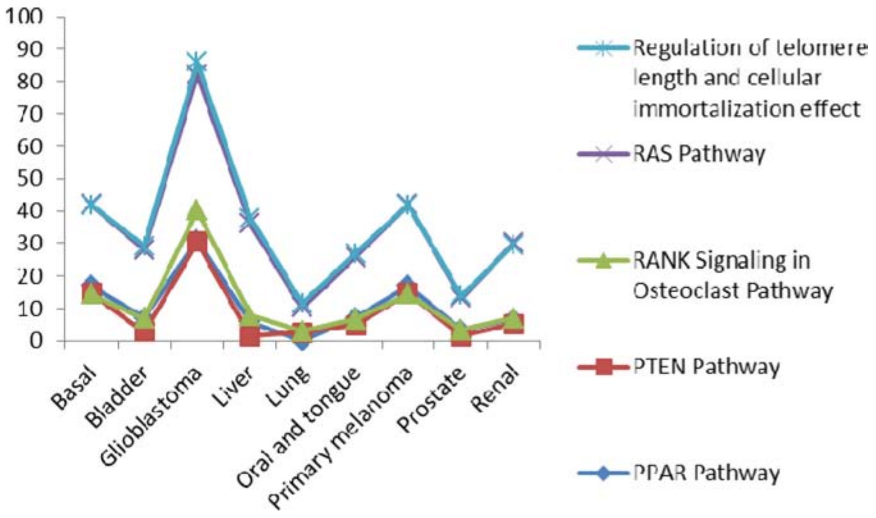

100
90
80
70
60
50
40
30
20
10
0
Basal
Bladder
Glioblastoma
Liver
Lung
Oral and tongue
Primary melanoma
Prostate
Renal
Regulation of telomere length and cellular immortalization effect
RAS Pathway
RANK Signaling in Osteoclast Pathway
PTEN Pathway
PPAR Pathway

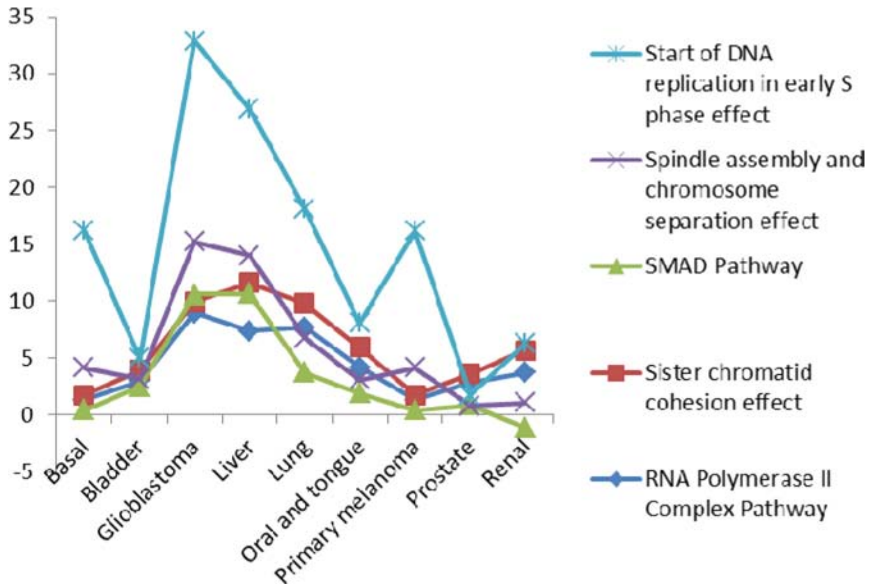

35
30
25
20
15
10
5
0
-5
Basal
Bladder
Glioblastoma
Liver
Lung
Oral and tongue
Primary melanoma
Prostate
Renal
Start of DNA replication in early S phase effect
Spindle assembly and chromosome separation effect
SMAD Pathway
Sister chromatid cohesion effect
RNA Polymerase II Complex Pathway

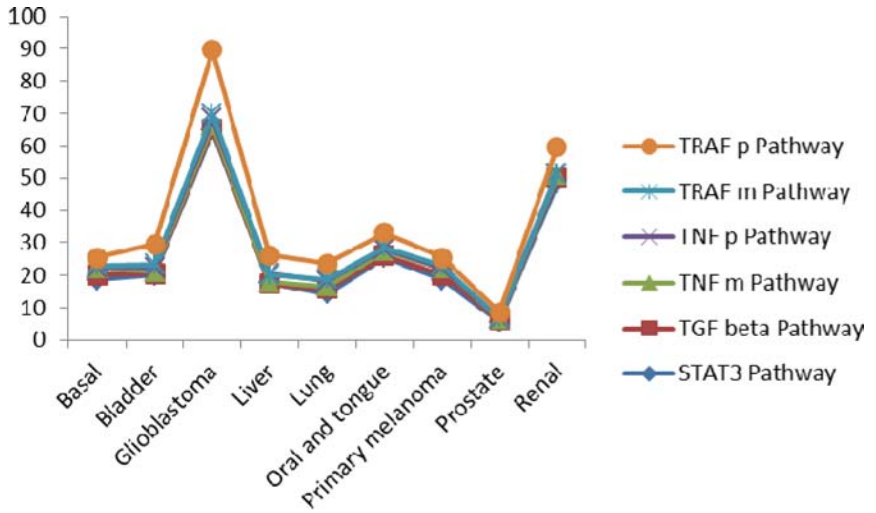

100
90
80
70
60
50
40
30
20
10
0
Basal
Bladder
Glioblastoma
Liver
Lung
Oral and tongue
Primary melanoma
Prostate
Renal
TRAF p Pathway
TRAF m Pathway
TNF p Pathway
TNF m Pathway
TGF beta Pathway
STAT3 Pathway

(*Continued*)

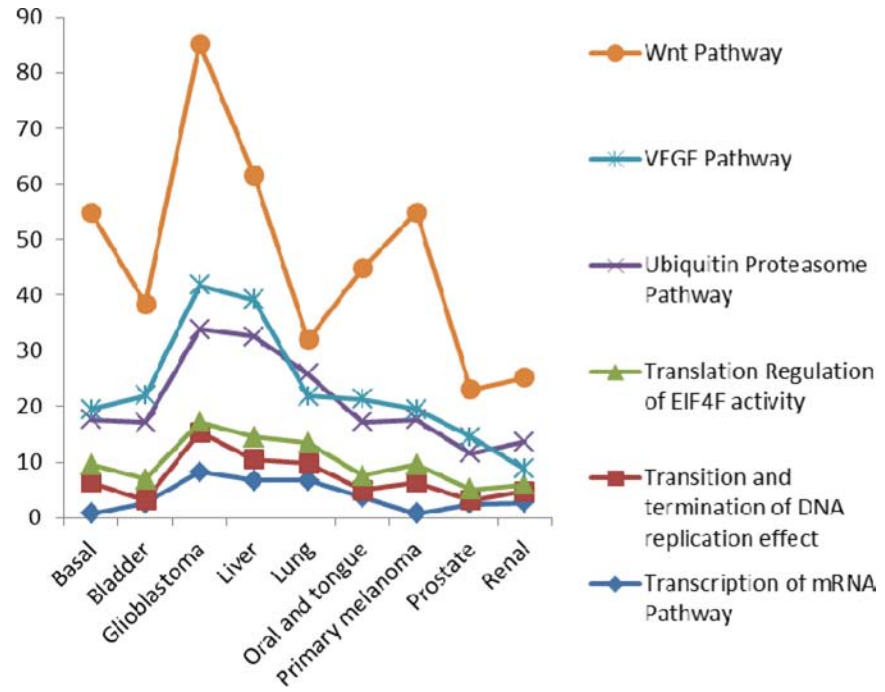
90
80
70
60
50
40
30
20
10
0
Basal
Bladder
Glioblastoma
Liver
Lung
Oral and tongue
Primary melanoma
Prostate
Renal
Wnt Pathway
VEGF Pathway
Ubiquitin Proteasome Pathway
Translation Regulation of EIF4F activity
Transition and termination of DNA replication effect
Transcription of mRNA Pathway
